# Functional Quality and Radical Scavenging Activity of Selected Watermelon (*Citrullus lanatus* (Thunb.) Mansfeld) Genotypes as Affected by Early and Full Cropping Seasons

**DOI:** 10.3390/plants12091805

**Published:** 2023-04-28

**Authors:** Imen Tlili, Riadh Ilahy, Leila Romdhane, Thouraya R’him, Hatem Ben Mohamed, Hatem Zgallai, Zouhair Rached, Muhammad Azam, Imen Henane, Mohamed Najib Saïdi, Zoltàn Pék, Hussein G. Daood, Lajos Helyes, Chafik Hdider, Marcello Salvatore Lenucci

**Affiliations:** 1 Laboratory of Horticulture, National Agricultural Research Institute of Tunisia (INRAT), University of Carthage, Menzah 1, Tunis 1004, Tunisia; 2 Arid and Oases Cropping Laboratory, Arid Regions Institute of Medenine, Medenine 4119, Tunisia; 3 Laboratory of Science and Agronomic Techniques, National Agricultural Research Institute of Tunisia (INRAT), University of Carthage, Menzah 1, Tunis 1004, Tunisia; 4 Laboratory of Rural Economy, National Agricultural Research Institute of Tunisia, University of Carthage, Menzah 1, Tunis 1004, Tunisia; 5 Pomology Laboratory, Institute of Horticultural Sciences, University of Agriculture, Faisalabad 38040, Pakistan; 6 Biotechnology and Plant Improvement Laboratory, Centre of Biotechnology of Sfax, Sfax 3018, Tunisia; 7 Laboratory of Horticulture, Faculty of Agricultural and Environmental Sciences, Horticultural Institute, Szent István University, 2100 Budapest, Hungary; 8 Dipartimento di Scienze e Tecnologie Biologiche ed Ambientali (Di.S.Te.B.A.), Università del Salento, Via Prov. le Lecce Monteroni, 73100 Lecce, Italy

**Keywords:** *Citrullus lanatus* (Thunb.) Mansfeld, citrulline, functional quality, growing seasons, radical scavenging activity

## Abstract

Growing conditions and seasonal fluctuations are critical factors affecting fruit and vegetable nutritional quality. The effects of two partially overlapping cropping seasons, early (ECS; January–May) and full (FCS; March–July), on the main carpometric traits and bioactive components of different watermelon fruits were investigated in the open field. Four watermelon genotypes, comprising of three commercial cultivars ‘Crimson Sweet’, ‘Dumara’, ‘Giza’, and the novel hybrid ‘P503 F1’, were compared. The carpometric traits varied significantly between genotypes. Soluble solids and yield were higher under FCS than ECS. The variation affecting colour indexes between the two growing seasons exhibited a genotype-dependent trend. The antioxidant components and radical scavenging activity of watermelon fruits were also significantly affected by differences in received solar energy and temperature fluctuations during the trial period. The average citrulline, total phenolics and flavonoid contents were 93%, 71% and 40% higher in FCS than in ECS. A genotype-dependent variation trend was also observed for lycopene and total vitamin C between cropping seasons. The hydrophilic and lipophilic radical scavenging activities of the pulp of ripe watermelon fruits of the different genotypes investigated varied between 243.16 and 425.31 µmol Trolox Equivalent (TE) of 100 g^−1^ of fresh weight (fw) and from 232.71 to 341.67 µmol TE of 100 g^−1^ fw in FCS and ECS, respectively. Our results, although preliminary, show that the functional quality of watermelon fruits is drastically altered depending on the environmental conditions that characterize the ECS and LCS.

## 1. Introduction

Consumption of watermelon (*Citrullus lanatus* (Thunb.) Mansfeld) throughout the Mediterranean basin is due, in large part, to its high nutritional value. Watermelon is of great economic importance, with world production estimated at 101 million tons [1]. Currently, China is the leading producer, followed by Turkey, India, Brazil, and Algeria. It has been reported that any fresh product with alleged or claimed health benefit is a functional food; therefore, watermelon can also be considered among functional foods [2,3]. Indeed, a plethora of bioactives such as carotenoids (lycopene), phenols, vitamins, and nonessential amino acids (citrulline and arginine) contribute to the functional quality of watermelon fruits [4,5,6]. These functional ingredients have the ability to scavenge free radicals and have been widely recognized to help prevent various human disorders, including cardiovascular disease, cancer, and age-related degenerative pathologies [7,8,9,10]. In addition, the low caloric value and health benefits of watermelon consumption make it a very popular fruit.

Cultural factors and seasonal fluctuations have a well-defined impact on the phytochemical composition and antioxidant capacity of most fruits and vegetables; however, research on the impact of environmental variables on the functional quality of watermelon fruits is still limited. Genotypic variability, agrotechnical measures, environmental conditions, and harvest and post-harvest management techniques all affect the amounts of health-beneficial phytochemicals and antioxidant activity in fruits and vegetables [11,12,13]. The functional components of watermelon have been investigated extensively. Genetic control has been reported to be the primary factor in determining the content of various antioxidants. Accordingly, wide variations have been reported in the contents, evaluated on a fresh weight (fw) basis of citrulline (0.5–7.21 mg g^−1^ fw), lycopene (35–112 mg kg^−1^ fw), β-carotene (0.1–2.1 mg kg^−1^ fw), vitamin C (111.3–260 mg RE kg^−1^ fw), total phenols (89.0–910 mg GAE kg^−1^ fw), and total flavonoids (111.3–260 mg RE kg^−1^ fw) [6,12,13,14,15,16,17,18,19,20,21,22,23].

Dorais et al. [24] revealed that temperature and light intensity have a direct impact on the quality characteristics of tomato fruits, such as texture, firmness, and sensory qualities, in addition to other research on several crops. Dumas et al. [11] examined the impact of weather and other pre-harvest conditions on the antioxidant content of tomato. Similar to tomato [3,25], changes in solar radiation and temperature can lead to significant changes in the antioxidant composition of watermelon fruits.

In a previous investigation, we showed unexploited variability in the antioxidant content of different watermelon genotypes depending on growing seasons; however, the study was focused only on the phenolic content [26].

Other authors have highlighted the effect of growing seasons on some quality traits of watermelon fruit, such as lycopene and total antioxidant power [27,28]. Therefore, a systematic analysis of the effect of cropping seasons on major watermelon quality traits is of great importance. Accordingly, the primary objective of this study was to examine changes in selected agronomical traits, in the contents of important bioactive molecules (citrulline, lycopene, total vitamin C, total phenolics, and flavonoid contents) and in the antioxidant activity of the ripe fruits of four watermelon cultivars as a function of two partially overlapping growth periods: the early cropping season (ECS; January–May) and the full cropping season (FCS; March–July).

## 2. Results and Discussion

### 2.1. Weather Conditions

**Figure 1:**
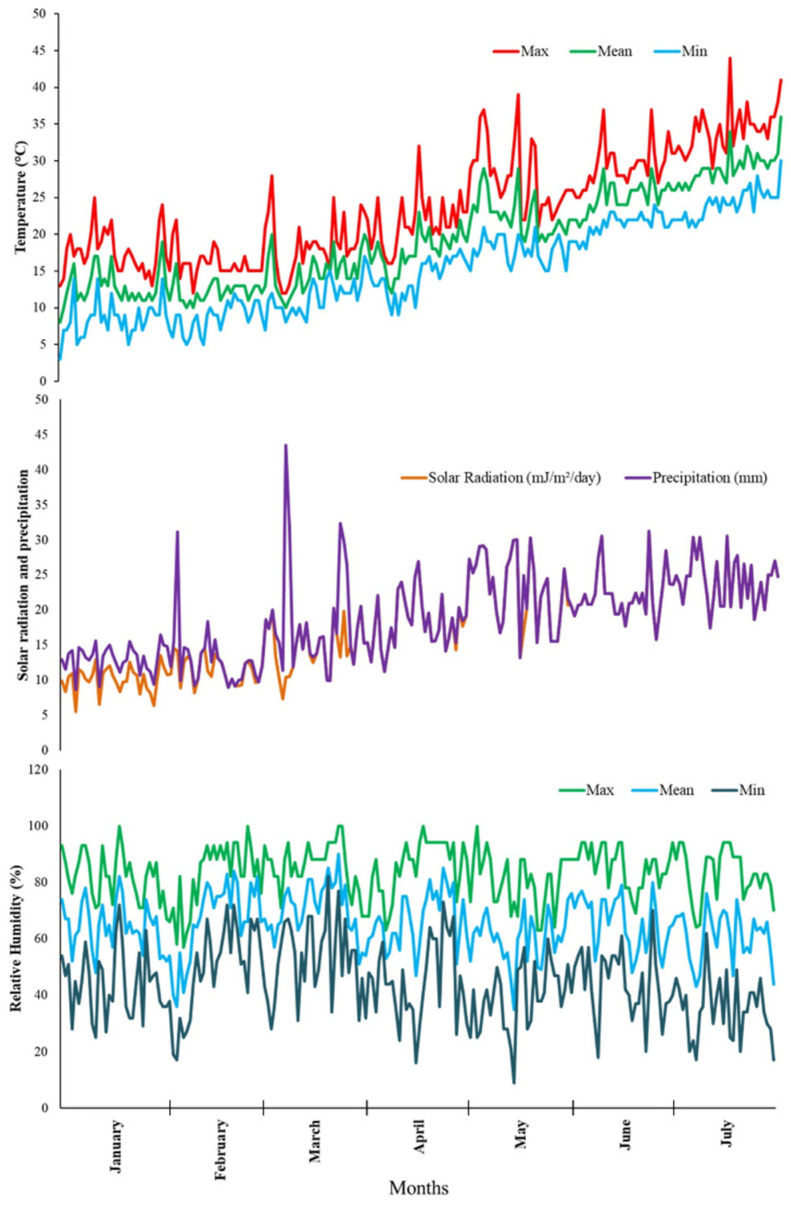
Temperature (°C), relative humidity (%), solar radiation (mJ m^−2^ day^−1^), and precipitation (mm) data recorded by the weather station of Teboulba–Monastir (35.637178; 10.957276). The reported data refer to 2016 and cover the whole watermelon growing seasons (January–July).

Data on temperature (°C), relative humidity (%), solar radiation (mJ m^−2^ day^−1^) and precipitation (mm) were recorded daily at the Teboulba Research and Experimental Station from the meteorological station of Monastir, Tunisia (Figure 1).

During the ECS, daily minimum and maximum temperatures ranged from 3 to 21 °C and 13 to 36 °C, respectively. Daily minimum and maximum relative humidity ranged between 17–82% and 62–100%, and the average solar radiation was about 8.8 mJ m^−2^ day^−1^. However, generally higher temperatures were recorded during the FCS, with daily minimum and maximum values ranging from 9 to 30 °C and from 12 to 44 °C, respectively. Solar radiation was also higher on average (13.8 mJ m^−2^ day^−1^) during FCS, while relative humidity remained almost unchanged with minimum and maximum values ranging from 16–82% to 63–100%, respectively. Precipitation ranged from 0 to 33.0 mm during the whole observation period. During the last 4 weeks prior to harvest, minimum and maximum temperatures were 18 and 28 °C, 24.3 and 34.4 °C, for the ECS and FCS, respectively, whereas mean solar radiation was 22.8 and 24.15 mJ m^−2^ day^−1^, respectively. Consequently, fruits grown under FCS received higher temperatures associated with higher solar radiation with respect to those grown under ECS. Precipitation ranged from 0 to 33.02 mm throughout the observation period. In the last 4 weeks before harvest, the minimum and maximum temperatures were 18 and 28 °C, 24.3 and 34.4 °C, respectively, for ECS and FCS, while the average solar radiation was 22.8 and 24.15 mJ m^−2^ day^−1^, respectively. Consequently, fruits grown under FCS received higher temperatures associated with higher solar radiation than those grown in ECS.

### 2.2. Carpometric Traits

The carpometric traits of the watermelon cultivars harvested at ECS and FCS are reported in Table 1.

Table 2 shows the averages of the carpometric determinations, the contents of the main bioactive molecules, and the radical scavenging activity of the four watermelon varieties in the two growing seasons.

#### 2.2.1. Marketable Yield

The marketable yield of watermelon cultivars grown during the ECS and FCS ranged from 6.0 to 8.0 kg plant^−1^ for ‘Crimson Sweet’, 6.08 to 7.92 kg plant^−1^ for ‘Giza’, 7.12 to 11.13 for ‘Dumara’, and 5.44 to 6.9 kg plant^−1^ for ‘P503 F1’ (Table 1). When averaged across the growing seasons, it varied from 6.56 to 9.13 kg plant^−1^. ‘Dumara’ had the highest yield, whereas ‘P503 F1’, ‘Giza’, and ‘Crimson Sweet’ were the least productive with no statistically significant differences among them. These results are in the same range as values previously published by our group [6,26].

Regardless of genotype, the results showed that marketable watermelon yields were significantly affected by the growing seasons (*p* < 0.05) (Table 2). In all cultivars studied, this parameter was higher in FCS than ECS. The mean values were 8.67 and 6.15 kg plant^−1^ in FCS and ECS, respectively. The difference in fruit yield was probably due to the fluctuation in weather conditions over the growing period, as reported in Figure 1. The data are consistent with those we have previously published on a larger number of watermelon cultivars [26]. Similarly, Susila et al. [27] found an increase in total yield, expressed as the number of fruits per plant for crop sown in December (FCS in Yderabad, India, where the research took place) compared to those sown early (October). The authors correlated the higher number of fruits and yield per plant under late versus early sown conditions with increased vine length, number of branches and flower, and fruit set.

#### 2.2.2. Average Fruit Weight

With regard to the average fruit weight, the results showed that it was significantly different among cultivars (*p* < 0.05) (Table 1). When mediated across cropping seasons, the fruits had an average weight between 4.62 (‘P503 F1’) and 6.98 kg (‘Dumara’), while ‘Crimson Sweet (6.01 kg) and ‘Giza’ (5.6 kg) showed an intermediate average weight.

The results also showed that the mean fruit fresh weight was affected by the growing seasons, regardless of cultivar (*p* < 0.05), varying from 5.08 to 6.5 kg in ECS and FCS, respectively (Table 2), corresponding to, approximatively, a 28% increase. In a previous preliminary study focusing on the effect of sowing date on some production traits of watermelon cultivars, Tlili et al. [26] noticed the absence of significant differences in mean fruit weight. Our results are also in agreement with those of Susila et al. [27], who reported a significant increase in watermelon fruit weight correlated with delay in sowing date. This fact was mainly ascribed to the high night temperatures during the ripening period of the crop sown in December, which could result in higher assimilate transport and sink activity, leading to a maximum/optimal increase in fruit weight compared to crop sown in October.

#### 2.2.3. Total Soluble Solids

The total soluble solids of ECS and FCS watermelon fruits ranged between 9.17 and 12.0 °Brix for ‘Crimson sweet’, 8.27–10.60 °Brix for ‘Giza’, 8.87–11.36 °Brix for ‘Dumara’ and 8.43–10.53 °Brix for ‘P503 F1’ (Table 1). Averaging the cropping seasons, the soluble solids varied from 9.43 to 10.58 °Brix in ‘Giza’ and ‘Crimson Sweet’, respectively. The latter being the cultivar with the highest value in both cropping seasons.

Regardless of genotype, watermelon soluble solids were also significantly affected by the growing seasons (*p* < 0.05; Table 2). The average values were higher in FCS (11.12 °Brix) than in ECS (8.68 °Brix). Again, this is likely due to an increase in the amount of solar radiation received by the plant during FCS, leading to an increase in the rate of photosynthesis and resulting in an accumulation of carbohydrates (mainly in the form of soluble sugars), which are major contributors to total solids, as previously reported for tomato fruits [29]. Furthermore, our results are in line with those of Susila et al. [27] who recorded the highest soluble solid and total sugar content in December-sown watermelons and correlated this with the frequency of higher temperatures during fruit ripening than that experienced by plants sown in October.

#### 2.2.4. Colour Indexes

Colour is one of the most important quality attributes for fresh fruits and vegetables. It is an attractive trait for consumers before purchasing fresh products [3]. Regardless of the growing seasons, significant variation (*p* < 0.05) in colour indices: lightness (L*), redness (a*), yellowness (b*), and (a*/b*) ratio was noticed among the studied watermelon cultivars (Table 1). The colour index (a*), which estimates the redness of watermelon flesh, was the highest in ‘P503 F1’ and ‘Giza’ when ECS and FCS data were considered, suggesting higher carotenoid accumulation in these genotypes.

When data from different cultivars were averaged, L*, a*, b* and a*/b* differed drastically between growing seasons (*p* < 0.05; Table 2). The colour of watermelon flesh was 11% lighter and redder in FCS (L* = 39.66; a* = 23.42) than ECS (L* = 35.89 a* = 21.16). Looking at the different cultivars, redness differed significantly between ECS and FCS for ‘Dumara’ and ’Giza’, but not for ‘Crimson Sweet’ and ‘P503 F1’, in which a* values appeared unaffected by the growing seasons. It is widely recognized that the final colour of watermelon flesh is strongly influenced by the levels and profile of carotenoids, especially lycopene, which is accumulated during ripening [3]. Similarly, with regard to yellowness (b*), the highest value was obtained under FCS (27.50) and the lowest under ECS (20.39). The a*/b* ratio, often used to assess the redness of fresh product, was higher under FCS only for ‘Crimson Sweet’ and ‘Dumara’; the average values of the four cultivars 51% were higher in FCS (1.34) than ECS (0.89). To our knowledge, the effects of growing conditions and seasonal fluctuations on colour indexes have not yet been studied for watermelon fruits, and these results are the first reported.

### 2.3. Functional Quality Attributes

The content of citrulline, total vitamin C, carotenoids, total phenols, and flavonoids in the four investigated watermelon cultivars grown in the ECS and FCS is reported in Table 3.

#### 2.3.1. Citrulline Content

The citrulline content of watermelon fruits grown under ECS and FCS ranged from 2.03 to 3.21 mg g^−1^ fw in ‘Crimson Sweet’, 1.23 to 2.3 mg g^−1^ fw in ‘Giza’, 0.47 to 1.88 mg g^−1^ fw in ‘Dumara’, and 1.40 to 2.51 mg g^−1^ fw in ‘P503 F1’ (Table 3).

Averaged across cropping seasons, the citrulline content was 1.17 to 2.62 mg g^−1^ fw in ‘Dumara’ and ‘Crimson Sweet’, respectively. Results from previously published studies reported a similar trend for citrulline ranging from 0.5 to 7.21 mg g^−1^ fw in different watermelon cultigens [12,14,15,16,17]. ‘Crimson Sweet’ was ranked among the most citrulline-rich cultivars, regardless of the growing seasons.

Statistically significant differences in citrulline concentration were also found between the growing seasons by averaging the values of the four cultivars, with a variation between 1.28 and 2.47 mg g^−1^ fw in ECS and FCS, respectively, representing a 93% increase (Table 2). Again, to the best of our knowledge, this is the first report on the effect of growing conditions and seasonal fluctuations on citrulline content.

#### 2.3.2. Carotenoid Content

The lycopene content of watermelon cultivars grown under ECS and FCS ranged from 44.50 to 56.53 mg kg^−1^ fw in ‘Crimson Sweet’, 72.5 to 107.7 mg kg^−1^ fw in ‘Giza’, 47.05 to 44.28 mg kg^−1^ fw in ‘Dumara’, and 77.83 to 116.13 mg kg^−1^ fw in ‘P503 F1’ (Table 3). Averaged over the cropping seasons, the lycopene content varied from 45.66 to 96.98 mg kg^−1^ fw in ‘Dumara’ and ‘P503 F1’, respectively. The obtained lycopene values are in line with those reported by several authors, ranging from 35 to 112 mg kg^−1^ fw in watermelon cultivars [6,18,19,20,21]. The results demonstrate that the lycopene concentration differed significantly between cropping seasons in most of the tested watermelon cultivars (*p* < 0.05), except for ‘Dumara’ (Table 2). The average lycopene concentration of the four cultivars was 34% greater in FCS (81.16 mg kg^−1^ fw) than ECS (60.48 mg kg^−1^ fw). ‘P503 F1’ and ‘Giza’ ranked among the best cultivar for lycopene content under both cropping seasons. This result might could be related to an increase in the amount of solar energy (light) intercepted by the plants, linked in turn to a temperature increase. Few studies have been conducted to investigate seasonal variations in the carotenoid content of watermelon fruits. The present results are consistent with those of Susila et al. [27], who reported a significant increase in lycopene for crop sown in December (FCS) compared to early (October) sowing, likely in relation to the effect of an average temperature increase in the FCS on carotenoids biosynthesis. Among studies on other crops, Heinonen et al. [30] demonstrated that lycopene concentrations in tomatoes purchased in local stores in Finland were relatively higher in summer (June to August) (3800–6600 µg 100 g^−1^ fw) than in winter (October to March) (2600–3100 µg 100 g^−1^ fw). Toor et al. [25], on the other hand, showed that the mean lycopene concentration of three tomato genotypes harvested in summer was 31% lower than at other times of the year. They noted that this was most likely due to temperatures below 12 °C, which substantially reduce lycopene biosynthesis, and temperatures above 32 °C, which completely stop this process, as described by Dumas et al. [11]. It is well recognized that the concentration of carotenoids is determined by the amount of photosynthetically active sunlight absorbed by the plant and by the temperature during fruit development [31].

The β-carotene level in watermelon cultivars grown under ECS and FCS ranged from 1.54 to 2.64 mg kg^−1^ fw in ‘Crimson Sweet’, 7.05 to 10.39 mg kg^−1^ fw in ‘Giza’, 1.95 to 2.28 mg kg^−1^ fw in ‘Dumara’ and 7.05 to 9.93 mg kg^−1^ fw in ‘P503 F1’ (Table 3). Averaged over the cropping seasons, the β-carotene content ranged from 2.08 to 8.72 mg kg^−1^ fw in ‘Crimson Sweet’ and ‘Giza’, respectively. The obtained values are lower than our previous data, which ranged from 0.1 to 2.1 mg kg^−1^ fw during the ripening of different watermelon genotypes grown in Southern Italy [6]. ‘P503 F1’ and ‘Giza’ ranked among the best cultivar for β-carotene content in both cropping seasons. Regardless of cultivars, watermelon fruits grown under FCS exhibited a 93% higher mean β-carotene content than in ECS (6.31 mg and 4.40 mg g^−1^ fw, respectively; Table 2).

The level of γ-carotene in watermelon cultivars grown under ECS and FCS ranged from 0.23 to 0.44 mg kg^−1^ fw ‘Crimson Sweet’, 0.58 to 1.00 mg kg^−1^ fw ‘Giza’, 0.22 to 0.25 mg kg^−1^ fw ‘Dumara’, and 0.84 to 0.91 mg kg^−1^ fw ‘P503 F1’ (Table 3). When averaged across growing seasons, it ranged from 0.24 to 0.87 mg kg^−1^ fw in ‘Dumara’ and ‘P503 F1’, respectively. To our knowledge, γ-carotene had not been detected previously in the same genotypes grown in an open field in Southern Italy. However, the presence of low amounts in our experiment could be due to ongoing climatic changes. Regardless of cultivar, γ-carotene levels were significantly different between growing seasons (*p* < 0.05) (Table 2), being 38% higher under FCS (0.65 mg kg^−1^ fw) than under ECS (0.47 mg kg^−1^ fw). To our knowledge, the effects of growing conditions and seasonal fluctuations on β-carotene and γ-carotene contents have not yet been investigated and these results are the first reported.

Light and circadian rhythms modulate the expression of most genes of the 2-C-methyl-D-erythritol 4-phosphate pathway and several genes of carotenoid synthesis and catabolism [32]. Furthermore, as previously mentioned, lycopene synthesis, and consequently, also that of the carotenoids downstream of the biosynthetic pathway, are strongly affected by low and high temperatures [11], making the weather conditions of the FCS more suitable for carotenoid accumulation than those of the ECS.

#### 2.3.3. Vitamin C Content

The total vitamin C content in watermelon cultivars varied depending on whether they were grown under ECS or FCS. In ‘Crimson Sweet’, the content ranged from 139.52 to 186.43 mg kg^−1^ fw, while in ‘Giza’, it ranged from 179.35 to 206.48 mg kg^−1^ fw. ‘Dumara’ had a range of 113.43 to 241.16 mg kg^−1^ fw, and ‘P503 F1’ of 138.01 to 114.94 mg kg^−1^ fw (Table 3). When averaged across growing periods, total vitamin C content varied from 126.48 to 192.92 mg kg^−1^ fw in P503 and Giza cultivars, respectively. These results are in conformity with previous reports where total vitamin C ranged from 38.2 to 576 mg kg^−1^ fw [4,6,13,21,33]. The observed variability might be ascribed to genotypic differences and applied agricultural practices as was outlined by Leskovar et al. [4].

The total vitamin C content in the P503 and Giza cultivars ranged from 126.48 to 192.92 mg kg^−1^ fw when averaged over the cropping season. These results are consistent with previous reports, which have shown a range of 38.2 to 576 mg kg^−1^ fw [4,6,13,21,33]. This variability in vitamin C content may be attributed to genotypic differences and the application of agronomic practices, as pointed out by Leskovar et al. [4].

In both cropping seasons, ‘Giza’ had the highest amount of vitamin C among all cultivars. However, overall vitamin C concentration varied significantly across cropping seasons regardless of cultivars (*p* < 0.05; Table 2). Apart from ‘P503 F1’, watermelon total vitamin C contents were higher in FCS for all cultivars. On average, the total vitamin C content was 142.58 mg kg^−1^ fw in ECS and 187.25 mg kg^−1^ fw in FCS, corresponding to an increase of 31%. These findings represent some of the earliest data on the variation affecting watermelon vitamin C content between cropping seasons.

According to Wadas and Mioduszewska [34], light intensity and temperature are the most critical pre-harvest elements in influencing the ultimate vitamin C content of vegetables. The amount and intensity of light during the growth seasons have a significant impact on the synthesis of ascorbic acid. Fruits that receive high sunshine light intensity synthesize and store a higher concentration of vitamin C than shaded fruits on the same plant.

Studies have shown that higher vitamin C content is generally detected in late-summer field-grown tomato fruits (250 to 350–400 mg kg^−1^ fw). Additionally, sparse foliage leading to higher solar radiation intercepted by fruits might increase vitamin C content [11]. Consequently, the suitable temperature and higher mean solar radiation in FCS compared to ECS might explain the higher accumulation of vitamin C content in most of the tested watermelon genotypes.

#### 2.3.4. Total Phenolics

Regardless of cropping seasons, the total phenolic content in watermelon cultivars varied from 118.12 to 171.18 mg GAE kg^−1^ fw for ‘Crimson Sweet’, 153.68 to 243.51 mg GAE kg^−1^ fw for ‘Giza’, 111.43 to 192.1 mg GAE kg^−1^ fw for ‘Dumara’, and 79.55 to 185.07 mg GAE kg^−1^ fw for ‘P503 F1’ (Table 3). On average, the values ranged from 132.31 to 198.60 mg GAE kg^−1^ fw for ‘P503 F1’ and ‘Giza’, respectively, across cropping seasons, which is consistent with previously reported data ranging from 89.0 to 910 mg GAE kg^−1^ fw [6,13,19,22,23]. ‘Giza’ was the cultivar with the highest phenolic content in both cropping seasons. There were significant differences in total phenolic levels between growing seasons (*p* < 0.05), ranging from 115.69 to 197.96 mg GAE kg^−1^ fw in ECS and FCS, respectively, regardless of the cultivar. When all genotypes were considered, the mean total phenolic content was 71% higher in samples from FCS than ECS (Table 2), in good agreement with our previous findings [26]. This difference could be attributed to the parallel increase in temperature and solar radiation that the plants received. Toor et al. [25] also reported a significant effect of cropping season on greenhouse-grown tomato fruit phytochemical composition, with 62% higher levels of phenolics in those harvested in the summer than in the spring season.

Environmental factors have a strong influence over genotypes in determining the content of phenolic compounds. Ilahy et al. [32] reported that thermal stress induces a massive phenolic accumulation in tomato and watermelon fruits by activating the biosynthetic and inhibiting the catabolic/oxidation pathways. This mechanism operates below 15 °C to overcome chilling stress in watermelon and above 35 °C in tomato to counteract heat stress and can be regarded as an acclimation response to extreme temperatures. This partly explains the higher accumulation of phenolic and flavonoids under FCS in all watermelon genotypes compared to ECS.

#### 2.3.5. Flavonoid Content

Despite variations in cropping seasons, the flavonoid concentration in watermelon cultivars was found to range from 180.47 to 235.27 mg RE kg^−1^ fw in ‘Crimson Sweet’, 183.89 to 277.08 mg RE kg^−1^ fw in ‘Giza’, 121.15 to 198.05 mg RE kg^−1^ fw in ‘Dumara’, and 187.26 to 232.21 mg RE kg^−1^ fw in ‘P503 F1’ (Table 3). After averaging flavonoid data across cropping periods, ‘Giza’ exhibited the highest levels (230.48 mg RE kg^−1^ fw). Statistically similar values were obtained for ‘P503 F1’ (209.73 mg RE kg^−1^ fw) and ‘Crimson Sweet’ (207.87 mg RE kg^−1^ fw), while the lowest value was recorded for ‘Dumara’ (159.60 mg RE kg^−1^ fw). These values align with previously reported data on numerous watermelon cultivars ranging from 111.3 to 260 mg RE kg^−1^ fw [6,13,20], with watermelon juice attaining 141 mg of quercetin equivalent kg^−1^ fw. Notably, ‘Giza’ exhibited the highest flavonoid amount in both cropping seasons.

Regardless of the cultivar, watermelon flavonoid contents were significantly affected by the growing seasons (*p* < 0.05) (Table 2). Watermelon flavonoid contents were higher in FCS across all cultivars. The average flavonoid content was 168.19 in ECS and 235.65 mg RE kg^−1^ fw in FCS, indicating a 40% increase. These findings are consistent with earlier work that reported a considerable increase in flavonoid content under FCS compared to ECS [26]. Like phenolics, Davies and Hobson [35] found that open-field-grown tomatoes, which receive more sunlight and UV radiation, contain more flavonoids than those produced under greenhouse conditions with artificial lighting.

### 2.4. Radical Scavenging Activity of the Hydrophilic and Lipophilic Fractions

The hydrophilic (HRSA) and lipophilic (LRSA) radical scavenging activities of the ripe fruits grown under ECS and FCS of the four investigated watermelon cultivars are reported in Table 4.

Table 4 indicates that ‘Crimson Sweet’ was the cultivar with the highest values for both HRSA and LRSA when data from the two different cropping seasons were averaged. The values were 425.31 µmol trolox equivalents (TE) 100 g^−1^ fw and 341.67 µmol TE 100 g^−1^ fw, respectively. These findings are consistent with previous studies, which reported slightly lower HRSA values ranging from 156.41 to 290.9 µmol TE 100 g^−1^ fw and similar LRSA values ranging from 195.23 to 467.9 µmol TE 100 g^−1^ fw [6,13,21].

When data from different cultivars were averaged, HRSA and LRSA differed drastically between cropping seasons (*p* < 0.05; Table 2). The average HRSA value was 255.04 µmol TE 100 g^−1^ fw in ECS and 348.33 µmol TE 100 g^−1^ fw in FCS, indicating a 37% increase. Similarly, the average LRSA value was 232.90 and 307.81 µmol TE 100 g^−1^ fw in ECS and FCS, respectively, corresponding to a 32% increase. These results provide a preliminary insight into the variability of HRSA and LRSA in watermelon cultivars in the two cropping seasons. However, Alan et al. [28] reported that ECS increased total antioxidant activity in comparison to LCS for the watermelon cultivar ‘Anthem’, by an average of 28.6%. Toor et al. [25] observed a significant positive effect of sun radiation and/or temperature on antioxidant activity. They found that the mean HRSA of three tomato cultivars was 39% higher in the summer than in the spring growing season. The increased lipophilic radical scavenging activity during the summer season could be attributed to the higher total phenolic content, which increased as sun radiation and temperature increased.

### 2.5. Correlation

As shown in Table 5 (and the related Supplementary Figure S1), colour indexes—particularly a*, b*, and the calculated a*/b* ratio—were significantly and highly correlated to flavonoid (r= 0.64, 0.69, −0.69, respectively), lycopene (r = 0.84, 0.82, −0.63, respectively), β-carotene (r= 0.89, 0.88, −0.68, respectively), and γ-carotene (r = 0.89, 0.91, −0.72, respectively) contents. However, the colour index L* was exclusively correlated to all assessed traits.

The results of this study indicate that citrulline is a major contributor to the antioxidant activity of watermelon samples, as evidenced by its positive and significant correlation with the contents of total phenolics (r = 0.54), total flavonoids (r = 0.80), HRSA (r = 0.80), and LRSA (r = 0.66). However, other compounds in both the hydrophilic and lipophilic fractions, such as lutein, prolycopene, xanthophylls, tocopherols, and others not assessed in this study, may also be responsible for the observed HRSA and LRSA values. To our knowledge, the effect of growing seasons on the correlation behaviour between agricultural and functional quality attributes has not been previously investigated. However, Ilahy et al. [36] observed similar trends when assessing the nutritional quality of different tomato cultivars grown in the open field in two cropping seasons.

## 3. Materials and Methods

### 3.1. Plant Culture and Growth Conditions

**Figure 2:**
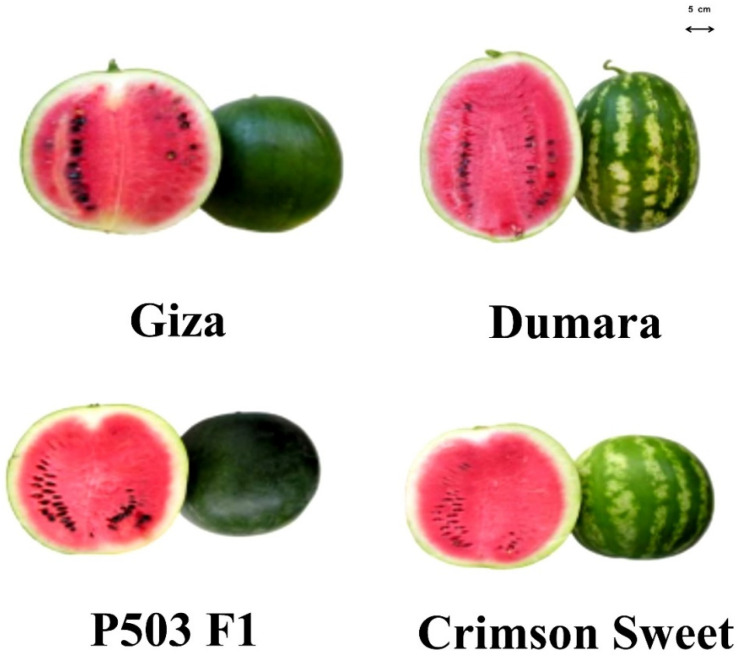
Longitudinal sections of the fruit of the four analysed watermelon cultivars.

Early cropping season ECS and full cropping season FCS field experiments were conducted at the Teboulba Research and Experimental Station in Monastir, Tunisia (35.637178, 10.957276). Four watermelon cultivars consisting of three commercial cultivars ‘Crimson Sweet’, ‘Dumara,’ ‘Giza’, and the recently developed hybrid ‘P503 F1’ were used (Figure 2). Seeds were sown in plug seedling trays at the middle of January and the beginning of March 2016 for ECS and FCS, respectively. Plants were transplanted to the soil on the 4th week of February under a low plastic tunnel, which was removed 1 month later, and on the 2nd week of April in open-field conditions for both ECS and FCS, respectively. In each growing season, randomized blocks were designed, which involved 3 replications with 10 plants per cultivar per replicate with a between-plant spacing of 150 cm and inter-row spacing of 125 cm. The ECS and FCS cultivars underwent similar cultural and agronomic practices throughout the investigation. To ensure optimal growth, an automated fertigation system was employed, utilizing synthetic chemical fertilizers containing 145 kg N ha^−1^, 140 kg P_2_O_5_ ha^−1^, and 210 kg K_2_O ha^−1^, which were applied twice weekly. In addition, other essential practices such as hand weeding and appropriate pesticide application for plant–pathogen control were implemented. Specifically, Imidacloprid (200 g L^−1^), Acetamipride (200 g L^−1^), and Abamectin (18 g L^−1^) were used once per cycle to combat aphids, thrips, and mites, respectively.

### 3.2. Watermelon Harvest and Sampling

Watermelon fruits were taken from completely mature commercial fruits. At least three injury-free watermelon fruits were handpicked at random from each cultivar and replicate. Harvests were performed at the end of May and the middle of the July for the first and second growing seasons, respectively. Only the heart portion (between the locular and the fruit centre) of each mature fruit was sampled for flesh. A minimum of one kilogram of the acquired sample was homogenized. At −80 °C, the homogenates were kept, frozen, and analysed within one week to avoid antioxidant loss occurring under freeze storage.

### 3.3. Carpometric Traits

Soluble solid content of the studied cultivars was expressed as the (°Brix) of fresh homogenate. The measurement was carried out by placing a drop of filtered watermelon juice on the prism of a hand-held digital refractometer with automatic temperature compensation (Atago 102 PR-100 NSG Precision Cells, Inc, Framing dale, NY). The flesh colour of watermelon fruits was assessed using a Minolta Chroma meter (CR-400, Minolta corp). The colour indexes L* (lightness), a* (redness), and b* (yellowness) were measured and the ratio (a*/b*) was calculated.

### 3.4. Functional Quality Attributes

#### 3.4.1. Determination of Citrulline Content

Citrulline was extracted as described by Rimando and Perkins-Veazie [14] with minor modification. Two hundred and fifty milligrams of the freeze-dried sample was used on triplicate. For 20 min, the sample was extracted with 10 mL of MeOH:6 M HCl (9:1) in a sonicating bath (DU 45 AGRO LAB). After filtering, the sample was washed twice with 5 mL of extraction solvent. The citrulline assay was performed by using the Colour Development Reagent (COLDER) according to the procedure of Knipp and Vasák [37]. A sample of 240 µL was added to 800 µL of COLDER (1 vol 80 mM diacetyl monoxime and 2.0 mM thiosemicarbazide; 3 vol 3 M H_3_PO_4_, 6 M H_2_SO_4_, and 2 mM NH_4_Fe[SO_4_]_2_). The mixture was heated at 95 °C for 15 min before being cooled to room temperature (10 min). After cooling to room temperature (10 min), the absorbance was read at 540 nm using a spectrophotometer (T60, PG Instruments Ltd., Leicestershire, UK). Citrulline content was expressed as (mg g^−1^ fw).

#### 3.4.2. Determination of Total Vitamin C

Total vitamin C content was determined on the homogeneous juice (3 replicates of 0.1 g for each cultivar), as was detailed by Tlili et al. [6]. The result was expressed in mg kg^−1^ fw.

#### 3.4.3. Determination of Carotenoid Content

Carotenoids were determined according to the protocol of Daood et al. [38] but slightly modified. Briefly, watermelon homogenate (2.5 g aliquots) was crushed in a crucible mortar with quartz sand. Subsequently, 20 mL of methanol was added for 1–2 min, and the upper layer was poured into an Erlenmeyer flask. A volume of 50 mL of dichloroethane was then mixed with 10 mL of methanol in a graduated cylinder and mixed softly before and after adding some distilled water drops. The mixture was filtrated in a separating funnel and allowed to evaporate at 70 °C until complete evaporation. A volume of 5 mL of analytical grade methanol was mixed with 5 mL of pigment eluents and poured into the same flask then mixed gently, sonicated, filtered using a 0.22 μm membrane syringe, and finally injected into the HPLC (Hitachi Chromaster, Tokyo, Japan) system consisting of a 5110 Pump, a 5210 Auto Sampler, a 5430 Diode Array detector, and a 5440 FL detector. The column effluents were detected at their maximum absorption wavelength. Lycopene, β- and ϒ-carotene were identified and quantified using authentic standards purchased from Sigma-Aldrich Ltd. (Budapest, Hungary). Peak areas of the different carotenoids detected were used to calculate their relative concentrations in the extracts.

#### 3.4.4. Determination of Total Phenols Content

The extraction of total phenol was conducted in accordance with the procedure of Tlili et al. [6] on three replicate samples of homogenate juice (0.3 g). Phenolic content was extracted at 4 °C with 50 µL of 37% HCl in 5 mL of 80% methanol under 300 rpm of continuous stirring for 2 h. Total phenol content was evaluated spectrophotometrically using the Folin–Ciocalteu method [39]. The absorbance was detected at 750 nm using a spectrophotometer (T60, PG Instruments Ltd., Leicestershire, UK). The results were given in milligrams of gallic acid equivalent per kilogram of watermelon fw (mg GAE kg^−1^ fw).

#### 3.4.5. Determination of Flavonoid Content

The flavonoid content of the homogeneous juice was evaluated on triplicate samples (0.3 g) [40]. A volume of 50 µL of methanolic extract was diluted with distilled water to a final volume of 0.5 mL and shaken vigorously with 30 µL of 5% NaNO_2_. After 6 min, 60 µL of 10% AlCl_3_ was added, followed by 200 µL of 1 M NaOH. Absorbance was measured at 510 nm, using a spectrophotometer (T60, PG Instruments Ltd., Leicestershire, UK). As rutin is the major glycosylated flavonoid in ripe red watermelon fruit and the only detected in the internal flesh [32], it was used as the standard for calibration and accurate estimation of total flavonoid concentrations, the values of which were expressed as mg of rutin equivalent (RE) kg^−1^ fw.

### 3.5. Determination of the Radical Scavenging Activity

The hydrophilic and lipophilic radical scavenging activity (HRSA and LRSA) were determined using the ABTS (2, 2′-Azino-Bis-3-Ethylbenzothiazoline-6-Sulfonic Acid di-ammonium salt) decolouration technique [41]. At 4 °C and 300 rpm of continual shaking, hydrophilic and lipophilic antioxidants were extracted from three replicated samples of 0.3 g homogeneous juice using 50 percent methanol or 50 percent acetone, respectively, for 12 h. The samples were centrifuged at 10,000× *g* for 7 min, and the various supernatants were measured. A PG 60 Instruments spectrophotometer was used to measure the antioxidant activity at 734 nm and values were reported as μM TE 100 g^−1^ fw.

### 3.6. Statistical Analysis

The experimental design for each growing season was a randomised complete block with four cultivars and three blocks/replicates. The analysis of variance was performed according to the General Linear Models (GLM) procedure developed by SAS (SAS Inst., V.6.1, Cary, NC, USA). The LSD test was applied to establish significant differences between means within growing seasons, and averages were also calculated and compared between growing seasons (considering all watermelon cultivars) as well as between cultivars (considering data of both growing seasons) with a confidence level of 95%. Results within each growing season are presented as the mean value ± standard deviation of three independent replicates (n = 3). Correlations were estimated using Pearson’s correlation coefficient (r). Scatter plots of bivariate correlations among TSS, total soluble solids; L*, lightness; a*, redness; b*, yellowness; a*/b*, a*/b* ratio; Citr, citrulline; TVC, total vitamin C; TPC, total phenolic compounds; TF, total flavonoids; Lyc, lycopene; β-Car, β-carotene; ϒ-Car, ϒ-carotene; HRSA, hydrophilic radical scavenging activity; and LRSA, lipophilic radical scavenging activity are presented in Supplementary Figure S1.

## 4. Conclusions

This study underscores the significant influence of the environmental growing conditions on the nutritional quality of watermelon fruits. Notably, a marked variation in health-promoting bioactive compounds and their corresponding antioxidant activity was observed among watermelon fruits, depending on the season of cultivation. Given that many bioactive compounds are key determinants of the colour, flavour, and taste of fruits and vegetables, it stands to reason that mid-summer-grown watermelon in the Mediterranean basin may offer greater sensory attributes and nutritional value than fruits grown under ECS.

All the watermelon genotypes tested performed better under FCS conditions in terms of horticultural traits and functional quality. Irrespective of the cropping seasons, the ‘Giza’ and ‘P503 F1’ genotypes accumulated higher levels of lycopene, total phenolics, and total vitamin C. However, ‘Crimson Sweet’ exhibited higher citrulline content associated with higher HRSA and LRSA values. While the precise impact of environmental conditions on the synthesis of the principal phytochemicals in watermelon remains unclear, this investigation yields valuable insight into the optimal growing period to attain the highest antioxidant potential that promotes good health and well-being. Additionally, considering the limited biological variability (just one year of sampling and two growing seasons/harvests) performed in this study, it provides an overview regarding the variability of horticultural traits and dynamics among antioxidant classes in different genotypes of watermelon affected by growing seasons.

It is noteworthy that this study only provides a preliminary overview of the variability of horticultural traits, dynamics among agronomic attributes, and antioxidant classes in different watermelon genotypes due to the limited biological variability resulting from one year of sampling and two cropping seasons/harvests. Therefore, further research is necessary to strengthen these findings, as they have significant scientific, commercial, and health implications related to the understanding of the underlying mechanisms.

## Figures and Tables

**Table 1:** Carpometric traits (marketable yield, average fruit weight, soluble solids, and colour indexes of watermelon cultivars harvested at ECS and FCS. Values represent the mean ± standard deviation of three independent sampling replicates (n = 3).

Cultivar	Cropping Season	Marketable Yield (kg Plant^−1^)	Average Fruit Weight (kg)	Soluble Solids (°Brix)	Colour Indexes			
					L*	a*	b*	a*/b*
Crimson Sweet								
	ECS	5.94 ± 0.13 b	5.12 ± 0.11 b	9.17 ± 0.14 b	37.00 ± 0.40 b	18.44 ± 0.64 a	10.31 ± 0.95 b	1.82 ± 0.18 a
	FCS	8.00 ± 0.14 a	6.90 ± 0.17 a	12.0 ± 0.12 a	39.17 ± 0.86 a	19.97 ± 0.50 a	24.40 ± 0.66 a	0.82 ± 0.03 b
	Mean	6.97 B	6.01 B	10.58 A	38.08 A	19.20 B	17.36 B	1.32 B
Giza								
	ECS	6.08 ± 0.27 b	4.97 ± 0.24 b	8.27 ± 0.18 b	32.90 ± 1.22 b	22.93 ± 0.55 b	29.53 ± 0.53 b	0.78 ± 0.03 a
	FCS	7.92 ± 0.21 a	6.27 ± 0.18 a	10.60 ± 0.12 a	35.23 ± 0.43 a	27.59 ± 0.68 a	34.66 ± 0.66 a	0.80 ± 0.01 a
	Mean	7.0 B	5.62 B	9.43 C	34.05 B	25.26 A	32.09 A	0.79 C
Dumara								
	ECS	7.12 ± 0.22 b	6.31 ± 0.47 b	8.87 ± 0.12 b	38.76 ± 1.50 b	19.16 ± 0.43 b	9.49 ± 0.18 b	2.02 ± 0.09 a
	FCS	11.13 ± 0.68 a	7.65 ± 0.32 a	11.36 ± 0.09 a	40.93 ± 0.32 a	21.52 ± 0.56 a	18.74 ± 1.08 a	1.16 ± 0.08 b
	Mean	9.13 A	6.98 A	10.12 B	39.85 A	20.34 B	14.12 C	1.59 A
P503 F1								
	ECS	5.44 ± 0.20 b	3.95 ± 0.16 b	8.43 ± 0.12 b	34.94 ± 0.35 b	24.12 ± 0.77 a	32.25 ± 0.56 a	0.75 ± 0.01 a
	FCS	6.90 ± 0.28 a	5.29 ± 0.20 a	10.53 ± 0.12 a	43.32 ± 0.38 a	24.63 ± 0.72 a	32.18 ± 1.47 a	0.77 ± 0.06 a
	Mean	6.57 B	4.62 C	9.48 C	39.13 A	24.38 A	32.22 A	0.76 C

L*, lightness; a*, redness; b*, yellowness; a*/b* ratio; ECS, early cropping season; FCS, full cropping season. Lower case and capital letters indicate, respectively, the mean separation within cropping seasons and within cultivars by the LSD test, *p* < 0.05.

**Table 2:** Average carpometric traits (marketable yield, fruit weight, soluble solids, and colour indexes), average functional quality attributes (citrulline, lycopene, β- and γ-carotene, total phenolics, flavonoids and total vitamin C contents), and average radical scavenging activity of the four watermelon cultivars in the ECS and FCS.

	Cropping Seasons	
	ECS	FCS
Carpometric traits		
Marketable yield (kg plant^−1^)	6.16 b	8.67 a
Average fruit weight (kg)	5.09 b	6.53 a
Total soluble solids (°Brix)	8.68 b	11.13 a
Colour indexes		
L*	35.89 b	39.66 a
a*	21.16 b	23.42 a
b*	20.39 b	27.50 a
a*/b*	0.89 b	1.34 a
Functional quality attributes		
Citrulline (mg g^−1^ fw)	1.29 b	2.48 a
Lycopene (mg kg^−1^ fw)	60.48 b	81.16 a
β-carotene (mg kg^−1^ fw)	4.40 b	6.31 a
γ-carotene	0.47 b	0.65 a
Total phenolics (mg GAE kg^−1^ fw)	115.70 b	197.97 a
Flavonoids (mg RE kg^−1^ fw)	168.19 b	235.65 a
Total vitamin C (mg kg^−1^ fw)	142.58 b	187.25 a
Radical scavenging activities TEAC (µmol TE 100 g−1 fw)		
HRSA	255.04 b	348.33 a
LRSA	232.90 b	307.81 a

L*, lightness; a*, redness; b*, yellowness; a*/b* ratio; ECS, early cropping season; FCS, full cropping season GAE, gallic acid equivalent; RE, rutin equivalent; TEAC, trolox equivalent antioxidant capacity; TE, trolox equivalents; HRSA, hydrophilic radical scavenging activity; LRSA, lipophilic antioxidant radical scavenging activity. In each row, values with the same letters are not significantly different (LSD test, *p* < 0.05).

**Table 3:** Citrulline, lycopene, β- and γ-carotene, total phenolics, flavonoids, and total vitamin C contents in ripe fruits of watermelon cultivars harvested at two different growing seasons. Values represent the mean ± standard deviation of three independent sampling replicates (n = 3).

Cultivar	Growing Season	Citrulline (mg g^−1^ fw)	Lycopene (mg kg^−1^ fw)	β-Carotene (mg kg^−1^ fw)	γ-Carotene (mg kg^−1^ fw)	Total Phenolics (mg EAG kg^−1^ fw)	Flavonoids (mg ER kg^−1^ fw)	Total Vitamin C (mg kg^−1^ fw)
Crimson Sweet								
	ECS	2.03± 0.03 b	44.50± 0.98 b	1.54 ± 0.06 b	0.23 ± 0.01 b	118.12 ± 1.13 b	180.47 ± 3.57 b	139.52 ± 5.92 b
	FCS	3.21± 0.01 a	56.53± 3.35 a	2.64 ± 0.10 a	0.44 ± 0.01 a	171.18 ± 3.41 a	235.27 ± 1.32 a	186.43 ± 5.36 a
	Mean	2.62 A	50.51 C	2.087 B	0.33 C	144.65 C	207.87 B	162.98 C
Giza								
	ECS	1.23± 0.00 b	72.55± 1.53 b	7.05 ± 0.43 b	0.58 ± 0.02 b	153.68 ± 3.19 b	183.88 ± 4.83 b	179.36 ± 1.59 b
	FCS	2.31 ± 0.02 a	107.70 ± 3.85 a	10.39 ± 0.50 a	1.00 ± 0.07 a	243.52 ± 3.90 a	277.08 ± 5.21 a	206.49 ± 4.94 a
	Mean	1.77 C	90.12 B	8.72 A	0.80 B	198.60 A	230.48 A	192.92 A
Dumara								
	ECS	0.47± 0.01 b	47.05± 0.72 a	1.95 ± 0.13 b	0.22 ± 0.01 a	111.43 ± 1.62 b	121.15 ± 0.48 b	113.44 ± 0.54 b
	FCS	1.88± 0.04 a	44.29± 1.25 a	2.28 ± 0.10 a	0.25 ± 0.01 a	192.10 ± 4.27 a	198.05 ± 3.61 a	241.17 ± 1.07 a
	Mean	1.17 D	45.66 C	2.12 B	0.24 D	151.77 B	159.60 C	177.30 B
P503 F1								
	ECS	1.40± 0.02 b	77.83± 3.34 b	7.05 ± 0.23 b	0.84 ± 0.04 a	79.55 ± 4.16 b	187.26 ± 5.75 b	138.01 ± 4.73 a
	FCS	2.51 ± 0.02 a	116.13± 2.1 a	9.93 ± 0.52 a	0.91 ± 0.02 a	185.07 ± 1.21 a	232.22 ± 2.59 a	114.94 ± 3.90 b
	Mean	1.95 B	96.98 A	8.49 A	0.87 A	132.31 D	209.74 B	126.48 D

GAE, gallic acid equivalent; RE, rutin equivalent; ECS, early cropping season; FCS, full cropping season. Lower case letters indicate mean separation within columns and the growing period by the LSD test, *p* < 0.05. Capital letters indicate mean separation among means within columns by the LSD test, *p* < 0.05.

**Table 4:** Hydrophilic (HRSA) and lipophilic (LRSA) radical scavenging activity (µmol TE 100 g^−1^) fw) in watermelon cultivars harvested at two different growing seasons. Values represent the mean ± standard deviation of three independent sampling replicates (n = 3).

Cultivar	Cropping Season	TEAC Assay (µmol TE 100 g^−1^ fw)	
		HRSA	LRSA
Crimson Sweet			
	ECS	313.25 ± 7.97 b	245.33 ± 3.03 b
	FCS	537.38 ± 15.2 a	438.01 ± 21.37 a
	Mean	425.31 A	341.67 A
Giza			
	ECS	255.27 ± 9.93 b	247.19 ± 2.40 b
	FCS	291.28 ± 4.7 a	230.06 ± 3.60 a
	Mean	273.27 B	238.62 C
Dumara			
	ECS	231.03 ± 1.64 b	231.95 ± 5.08 b
	FCS	255.29 ± 0.83 a	304.90 ± 4.22 a
	Mean	243.16 C	268.42 B
P503 F1			
	ECS	220.60 ± 3.63 b	207.15 ± 4.80 b
	FCS	309.36 ± 13.01 a	258.27 ± 8.31 a

TEAC, trolox equivalent antioxidant capacity; TE, trolox equivalents; HRSA, hydrophilic antioxidant radical scavenging activity; LRSA, lipophilic radical scavenging activity; ECS, early cropping season; FCS, full cropping season. Lower case letters indicate mean separation within columns and the growing period by the LSD test, *p* < 0.05. Capital letters indicate mean separation among means within columns by the LSD test, *p* < 0.05.

**Table 5:** Pearson correlation coefficients between all investigated traits calculated based on the values of two cropping seasons. n (sample size) = 24.

Trait	TSS	L*	a*	b*	a*/b*	Citr	TVC	TPC	Flav	Lyc	β-Car	ϒ-Car	HRSA	LRSA
**TSS**	1	0.57 **	0.53 ^ns^	0.10 ^ns^	−0.26 ^ns^	0.78 **	0.52 **	0.68 **	0.60 **	0.56 ^ns^	−0.07 ^ns^	−0.018 ^ns^	0.67 **	0.75 **
L*		1	−0.18 ^ns^	−0.19 ^ns^	0.12 ^ns^	0.33 ^ns^	−0.11 ^ns^	0.20 ^ns^	0.07 ^ns^	0.03 ^ns^	−0.15 ^ns^	−0.16 ^ns^	0.24 ^ns^	0.32 ^ns^
a*			1	0.84 **	0.65 **	0.16 ^ns^	0.21 ^ns^	0.50 *	0.64 **	0.84 **	0.89 **	0.89 **	−0.23 ^ns^	−0.33 ^ns^
b*				1	0.92 **	0.35 ^ns^	0.18 ^ns^	0.40 *	0.69 **	0.82 **	0.88 **	0.91 **	0.03 ^ns^	0.07 ^ns^
a*/b*					1	0.48 *	−0.33 ^ns^	−0.40 *	−0.69 **	−0.63 **	−0.68 **	−0.72 **	−0.20 ^ns^	−0.19 ^ns^
**Citr**						1	0.31 ^ns^	0.54 **	0.80 **	0.29 ^ns^	0.16 ^ns^	0.27 ^ns^	0.80 **	0.66 **
**TVC**							1	0.61 **	0.45 *	−0.14 ^ns^	−0.57 ^ns^	−0.04 ^ns^	0.17 ^ns^	0.37 ^ns^
**TPC**								1	0.77 **	0.46 *	0.42 *	0.35 ^ns^	0.27 ^ns^	0.28 ^ns^
**Flav**									1	0.65 **	0.60 **	0.66 **	0.44 *	0.28 ^ns^
**Lyc**										1	0.95 **	0.93 **	−0.07 ^ns^	−0.29 ^ns^
**β-Car**											1	0.94 **	−0.20 ^ns^	−0.38 ^ns^
**ϒ** **-Car**												1	−0.09 ^ns^	−0.29 ^ns^
**HRSA**													1	0.89 **
**LRSA**														1

TSS, total soluble solids; L*, lightness; a*, redness; b*, yellowness; a*/b*, a*/b* ratio; Citr, citrulline; TVC, total vitamin C; TPC, total phenolic compounds; TF, total flavonoids; Lyc, lycopene; β-Car, β-carotene; ϒ-Car, ϒ-carotene; HRSA, hydrophilic radical scavenging activity; LRSA, lipophilic radical scavenging activity; ns, non-significant. *, ** = significant at *p* < 0.05 or *p* < 0.01, respectively.

## Data Availability

Not Applicable.

## Supplementary Materials

The following supporting information can be downloaded at: https://www.mdpi.com/article/10.3390/plants12091805/s1, Pearson correlation matrix (A) and scatter plots of bivariate correlations (B) for all investigated traits calculated based on values of two cropping seasons.
