# Supplementary material for: Functional Quality and Radical Scavenging Activity of Selected Watermelon (Citrullus lanatus (Thunb.) Mansfeld) Genotypes as Affected by Early and Full Cropping Seasons
Source: Plants (Basel). 2023 Apr 28;12(9):1805. doi: 10.3390/plants12091805 (PMC10181218; doi:10.3390/plants12091805)

Supplementary Figure S1. Pearson correlation matrix **(A)** and scatter plots of bivariate correlations **(B)** for all investigated traits calculated based on values of two cropping seasons. n (sample size) = 24. TSS, total soluble solids; L*, lightness; a*, redness; b*, yellowness; a*/b*, a*/b* ratio; Citr, citrulline; TVC, total vitamin C; TPC, total phenolic compounds; TF, total flavonoids; Lyc, lycopene; β-Car, β-carotene; Ƴ-Car, Ƴ-carotene; HRSA, hydrophilic radical scavenging activity; LRSA, lipophilic radical scavenging activity. In A, blueish tones indicate higher positive correlations, whereas reddish tones point towards negative correlations. Larger circle diameters denote higher modules of the correlation coefficient (r). *, **, *** = significant at $P < 0.05$, $P < 0.01$ or $P < 0.001$, respectively.

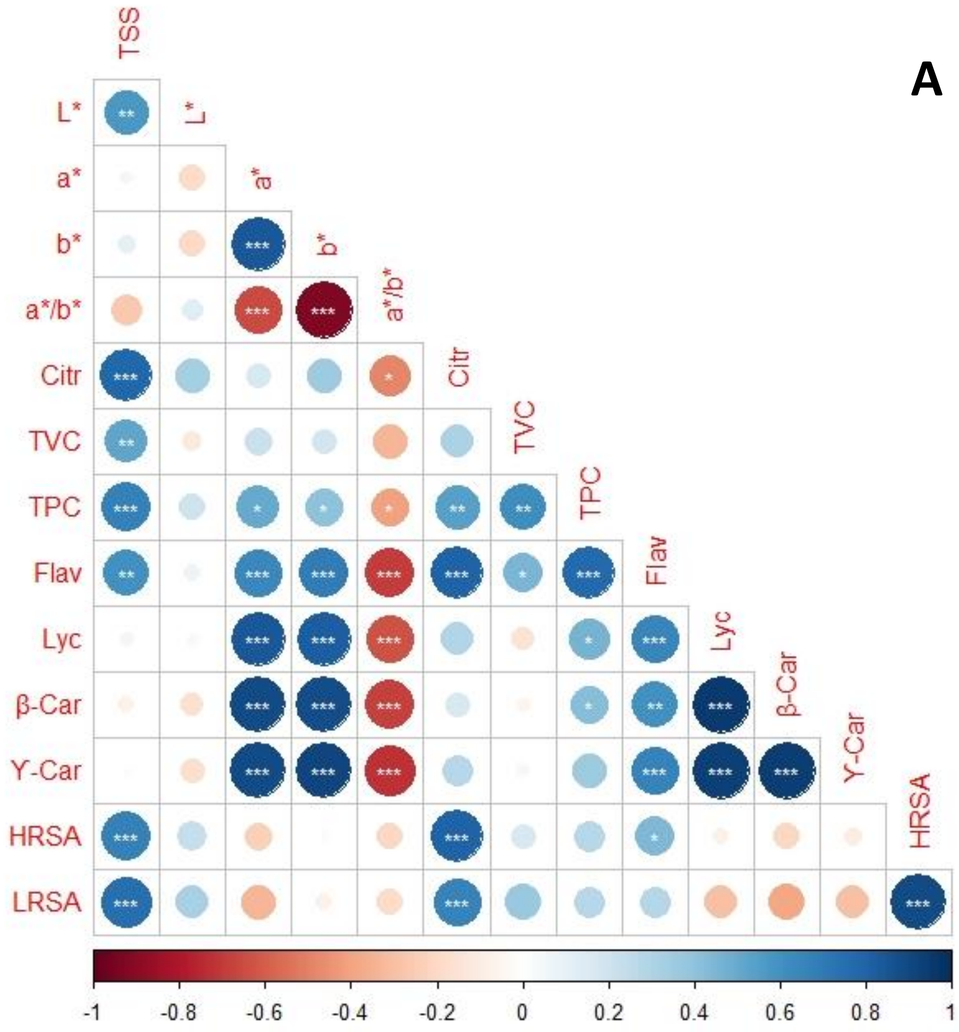

B

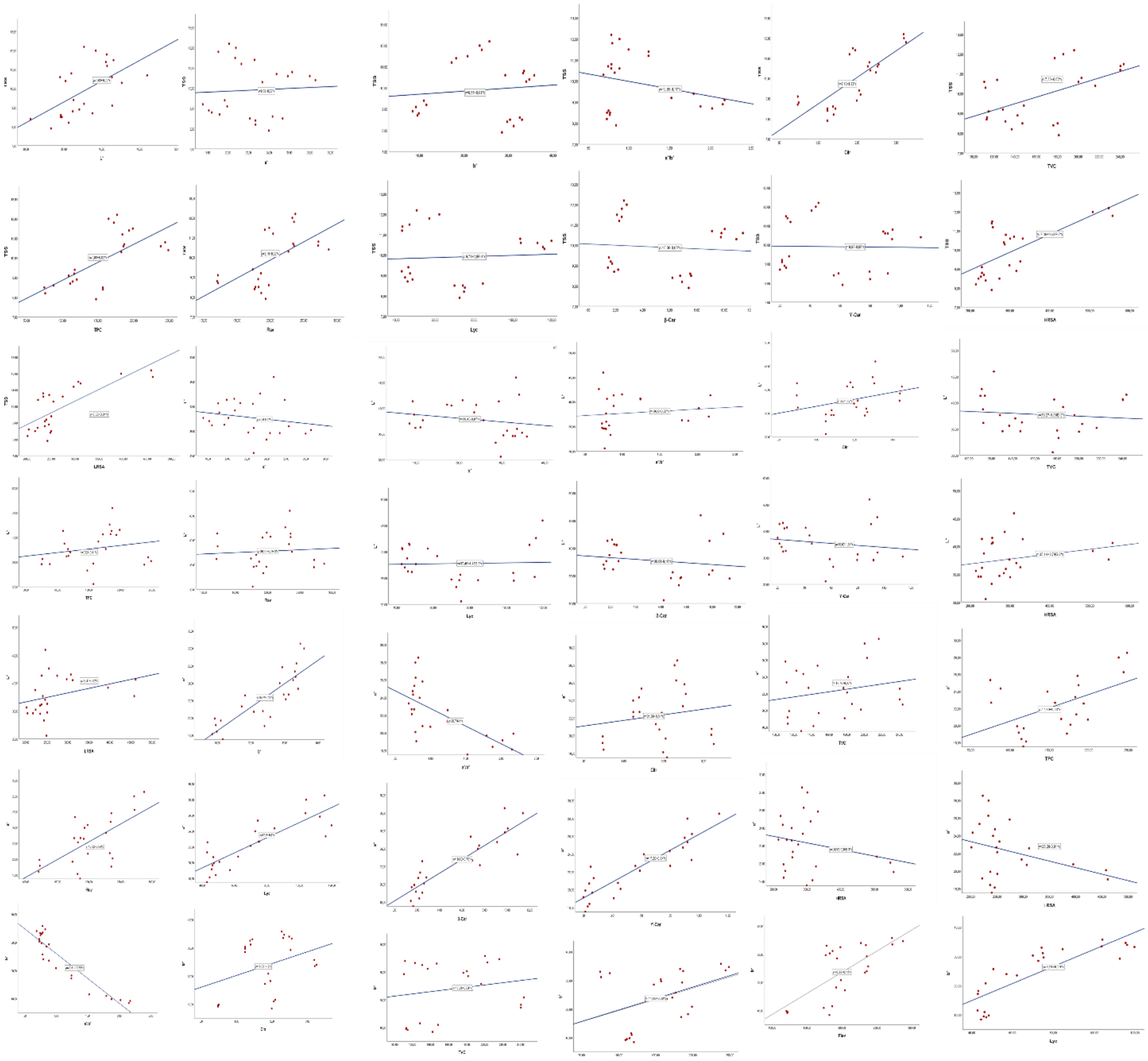

Supplement: Supplementary file 1 [file plants-12-01805-s001.zip › plants-2282996-supplementary.pdf]
